# Reply to Liu: The disassembly of the actin cytoskeleton is an early event during NETosis

**DOI:** 10.1073/pnas.2015951117

**Published:** 2020-09-15

**Authors:** Hawa Racine Thiam, Siu Ling Wong, Rong Qiu, Mark Kittisopikul, Amir Vahabikashi, Anne E. Goldman, Robert D. Goldman, Denisa D. Wagner, Clare M. Waterman

**Affiliations:** ^a^Cell and Developmental Biology Center, National Heart, Lung, and Blood Institute, National Institutes of Health, Bethesda, MD 20892;; ^b^Lee Kong Chian School of Medicine, Nanyang Technological University, Singapore 308232;; ^c^Department of Cell and Molecular Biology, Northwestern University Feinberg School of Medicine, Chicago, IL 60611;; ^d^HHMI, Ashburn, VA 20147;; ^e^Program in Cellular and Molecular Medicine, Boston Children's Hospital, Boston, MA 02115;; ^f^Department of Pediatrics, Harvard Medical School, Boston, MA 02115;; ^g^Division of Hematology/Oncology, Boston Children's Hospital, Boston, MA 02115

We used quantitative, high-resolution live-cell imaging of neutrophil-like human cells (dHL60) and mouse and human blood-derived polymorphonuclear neutrophils (PMNs) stimulated with ionomycin, lipopolysaccharide (LPS), or *Candida albicans* to identify 13 cellular events occurring during neutrophil extracellular trap formation (NETosis) (1). Following thousands of single cells over time showed that these events occurred in a specific order, with actin disassembly preceding all other cellular events observed, indicating that actin disassembly is an early event in NETosis (1).

In their letter to the editor, Liu challenges our conclusion (2). Liu uses flow cytometry of PMA-stimulated cells stained with “RFP-labeled phalloidin” to show that, at the population level, phalloidin fluorescence increases 60 min after addition of PMA and returns to basal level at 180 min, concluding that actin disassembly is not an early event of NETosis (2).

The points below support our published contention that actin disassembly is an early event in NETosis.

**Fig. 1.**
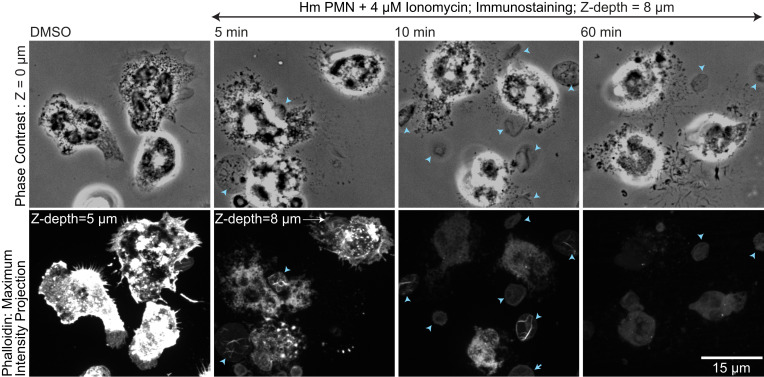
Human blood polymorphonuclear neutrophils (Hm PMN) were plated on coverslips, stimulated with 4 μM ionomycin or vehicle control (dimethyl sulfoxide [DMSO]), and fixed at different time points after stimulation (noted in images). Cells were stained with phalloidin-Alexa fluor 694 and imaged by phase contrast (*Top*) and spinning-disk confocal microscopy (*Bottom*). *Bottom* images (phalloidin) represent maximum intensity projections of Z stacks (step size, 0.2 μm; total Z-depth, 5 μm [DMSO] or 8 μm [stimulated, to account for the increase in cell height after stimulation]). Double-arrow-headed line above images indicates cells that are in the presence of ionomycin. Cyan arrows indicate red blood cells (RBC). Note the presence of actin bundles in RBCs 5 and 10 min after the addition of ionomycin. Note the global decrease in phalloidin signal in Hm PMNs between before and after ionomycin stimulation in cells that shed microvesicles.

1) Published papers support our findings and contradict those of Liu: a) Neubert et al. (3) showed a decrease in SiR-actin staining by live microscopy within 60 min after PMA treatment. b) Metzler et al. (4) showed, by Western blotting, the degradation of actin 30 min after stimulation with *C. albicans.* c) Sollberger et al. (5) showed a lack of phalloidin staining by microscopy 120 min after PMA addition.
2) Liu’s flow cytometry analysis requires cells in suspension, while ours and other studies report actin disassembly during NETosis of adherent cells (1, 3, 5). Neutrophils in situ adhere via integrins to extracellular matrix, other cells, or clots, and integrin engagement to an immobile ligand contributes to NETosis (6, 7). While PMA induces integrin activation, the absence of immobile ligands in suspension would fail to initiate integrin-mediated activation of actin regulatory pathways (8) during NETosis.
3) Liu defines time 0 as the addition of PMA stimulant, while we define time 0 as the first event visible by differential interference contrast microscopy (microvesicle shedding) after addition of stimulant marking entry into NETosis. We found that the time from stimulation to microvesicle shedding was highly variable, occurring between 0 min and 211 min (mean = 15±33) after ionomycin addition, and not all stimulated cells entered or completed NETosis (1). Thus, the addition of stimulant is distinct from the beginning of NETosis. Once microvesicles shed, NETosis events occur in a specific sequence with consistent timing. Independent of whether a cell sheds microvesicles at 5 min or 200 min after stimulation, shedding is always preceded by actin disassembly (1). Thus, actin disassembly is not an early event after stimulation, but is an early event in NETosis.
4) We verified actin disassembly using three different neutrophil types and two different stimulants, and actin filament depolymerization is visualized using four different actin probes [GFP-actin (9), F-tractin-mApple (9, 10), SiR-actin (11), and fluorescent phalloidin (Fig. 1)]. In our live-cell analysis, actin disassembly was defined as the first time point where the fluorescent actin probe concentrated in lamellipodia, the cortex, and uropods was reduced and became homogeneous throughout the cell, indicating a transition from filamentous to disassembled actin.
